# Potassium Channel-Associated Bioelectricity of the Dermomyotome Determines Fin Patterning in Zebrafish

**DOI:** 10.1534/genetics.120.303390

**Published:** 2020-06-16

**Authors:** Martin R. Silic, Qiuyu Wu, Brian H. Kim, Greg Golling, Kenny H. Chen, Renata Freitas, Alexander A. Chubykin, Suresh K. Mittal, GuangJun Zhang

**Affiliations:** * Department of Comparative Pathobiology, Purdue University, West Lafayette, Indiana 47907; † Department of Biological Sciences, Purdue University, West Lafayette, Indiana 47907; ‡ Life Science Department, Taft College, California 932608; § Institute for Innovation and Health Research (i3S), Instituto de Ciências Biomédicas Abel Salazar/University of Porto, Portugal 4200-135; ** Purdue Institute for Integrative Neuroscience, Purdue University, West Lafayette, Indiana 47907; †† Purdue University Center for Cancer Research, Purdue University, West Lafayette, Indiana 47907; ‡‡ Purdue Institute of Inflammation, Immunology, and Infectious Diseases (PI4D), Purdue University, West Lafayette, Indiana 47907

**Keywords:** potassium channel, *kcnj13/kir7.1*, bioelectricity, somite, dermomyotome, long-fin, patterning, CRISPR, zebrafish

## Abstract

The roles of bioelectric signaling in developmental patterning remain largely unknown, although recent work has implicated bioelectric signals in cellular processes such as proliferation and migration. Here, we report a mutation in the inwardly rectifying potassium channel (*kir*) gene, *kcnj13/kir7.1*, that causes elongation of the fins in the zebrafish insertional mutant Dhi2059. A viral DNA insertion into the noncoding region of *kcnj13* results in transient activation and ectopic expression of *kcnj13* in the somite and dermomyotome, from which the fin ray progenitors originate. We made an allele-specific loss-of-function *kcnj13* mutant by CRISPR (clustered regularly interspaced short palindromic repeats) and showed that it could reverse the long-finned phenotype, but only when located on the same chromosome as the Dhi2059 viral insertion. Also, we showed that ectopic expression of *kcnj13* in the dermomyotome of transgenic zebrafish produces phenocopies of the Dhi2059 mutant in a gene dosage-sensitive manner. Finally, to determine whether this developmental function is specific to *kcnj13*, we ectopically expressed three additional potassium channel genes: *kcnj1b*, *kcnj10a*, and *kcnk9*. We found that all induce the long-finned phenotype, indicating that this function is conserved among potassium channel genes. Taken together, our results suggest that dermomyotome bioelectricity is a new fin-patterning mechanism, and we propose a two-stage bioelectricity model for zebrafish fin patterning. This ion channel-regulated bioelectric developmental patterning mechanism may provide with us new insight into vertebrate morphological evolution and human congenital malformations.

## Abstract (precis)

It has long been recognized that the morphological complexity of vertebrates is established by spatially- and temporally-regulated cell signaling. For decades, studies of the molecular mechanisms....

MORPHOLOGICAL diversity in living organisms is exhibited by substantially varied forms and sizes of different body parts (Lecuit and Le Goff 2007). One of the fundamental questions in developmental biology is how this diversity is created through whole-body and regional patterning, a process that occurs in early embryonic development (Takahashi *et al.* 2001; Lander 2011). Patterning includes many developmental and cellular events such as axial patterning, segmentation, tissue formation, organ size determination, cell proliferation, cell death, and differentiation (Salazar-Ciudad *et al.* 2003; Lander 2011). Furthermore, many human congenital diseases are due to errors during embryonic development.

Understanding the mechanisms that pattern embryos remains a central challenge in developmental biology. Historically, such efforts have focused on identifying specialized signaling regions, the cells that produce and respond to these signals, and, ultimately, the nature of the signaling molecules themselves (Rogers and Schier 2011; Briscoe and Small 2015). Recent evidence suggests that bioelectricity, endogenous electrical signaling mediated by ion channels and pumps on the cell membrane, plays an important role in embryonic development. Bioelectricity has expanded the paradigm of cell signaling mechanisms (Levin 2014a). One of the major contributors to bioelectricity is potassium due to its high intracellular concentration. In humans, 78 potassium channels are classified into four categories: voltage-gated potassium channels (Kv), calcium-activated potassium channels (KCa), two-pore domain potassium channels (K2P), and inwardly rectifying potassium channels (Kir). The Kir channels possess unusual characteristics, as they do not have a voltage-sensing domain and conduct K^+^ more easily into, rather than out of, the cell under physiological conditions. Thus, they play important roles in maintaining resting membrane potential and other cellular bioelectric properties (Hibino *et al.* 2010). Kir channels are tetramers, and each subunit has two transmembrane (TM1 and TM2) regions and a pore-forming loop (H5/P-loop) (Hibino *et al.* 2010), which are critical for the potassium conductivity. So far, the most well-studied systems of bioelectricity and potassium channels are excitable cells such as neurons and myocytes. Recent studies have suggested that bioelectricity also plays important roles in nonexcitable cells, specifically in epithelial cells during vertebrate embryogenesis and wound healing (Zhao *et al.* 2006; Levin 2014b). For example, *Kcnj2* (*Kir2.1*) was found to be required for craniofacial and digit patterning in mice (Dahal *et al.* 2012), and *KCNJ2* mutations cause clinodactyly in humans (Plaster *et al.* 2001). Similarly, the Kir channel *KCNJ13* (*Kir7.1*) has been reported to be linked to snowflake vitreoretinal degeneration and Leber congenital amaurosis in humans (Hejtmancik *et al.* 2008; Sergouniotis *et al.* 2011), cleft palate and tracheal tubulogenesis in mice (Villanueva *et al.* 2015; Yin *et al.* 2018), and pigmentation pattern changes in zebrafish (Iwashita *et al.* 2006).

Development of the appendicular skeleton, fish fins, and tetrapod limbs provides an ideal model for understanding patterning mechanisms in vertebrates (Zeller *et al.* 2009). Though fish fins and tetrapod limbs share a common evolutionary origin, their distal portions have experienced independent morphological diversification (Clack 2009). Tetrapod limbs have further elaboration of the distal endoskeleton, forming digits instead of dermal fin rays and scales, while fish fins have retained fin rays distally connected to the endoskeleton, (Grandel and Schulte-Merker 1998). Thus, during the fish fin–tetrapod limb transition, the general pattern was concomitantly expanding the endochondral bone and distally reducing the dermoskeleton (fin rays). This culminated with the evolution of digits in stem groups of tetrapods (Boisvert *et al.* 2008; Shubin *et al.* 2009). The dermoskeleton of fish fins was long thought to be derived from neural crest cells (Smith *et al.* 1994). However, recent findings have shown that somite cells contribute to fin ray development, suggesting a common mesodermal developmental origin for both fin rays and digits (Lee *et al.* 2013a,b; Mongera and Nusslein-Volhard 2013; Shimada *et al.* 2013). These distal fin and limb structures have also been found to share regulatory control modules that govern transcription of 5′ HoxD genes (Nakamura *et al.* 2016; Lalonde and Akimenko 2018). By contrast, the mechanisms that control the patterning of fin rays, which underwent extensive morphological diversification in fish, is largely unknown. Forward genetic screens in zebrafish have identified fin ray anomalies associated with mutations in genes encoding the potassium channel *kcnk5b*, the gap junction protein *connexin 43* (*cx43*), a potassium chloride cotransporter (*kcc4a*), and the teleost-specific gene *Rapunzel* (Green *et al.* 2009; Sims *et al.* 2009; Perathoner *et al.* 2014; Lanni *et al.* 2019). Although the developmental mechanisms underlying the fin ray phenotypes are not fully understood, these mutants raise the critical role of bioelectricity in zebrafish fin patterning.

Here, we report a zebrafish fin elongation mutant, *kcnj13^Dhi2059^*, identified from a large-scale retroviral insertional mutagenesis screen (Golling *et al.* 2002; Amsterdam *et al.* 2004). In this mutant, retroviral DNA sequences were inserted into the protein noncoding fifth exon of the *kcnj13* gene. This insertion leads to *kcnj13* being ectopically and transiently activated in the somites during early development. The *kcnj13* CRISPR (clustered regularly interspaced short palindromic repeats) mutations *in cis* to the Dhi2059 insertion result in reversion to wild-type fin phenotypes, *i.e.*, the elongated fins of Dhi2059 can be blocked by loss-of-function of the *kcnj13* allele linked with the viral insertion, but not by the nonlinked *kcnj13* isoallele in Dhi2059. In addition, the elongated fins can be phenocopied by ectopically expressing *kcnj13* in the dermomyotome of transgenic fish Tg(*pax3a-5.4k:kcnj13-IRES-EGFP*) in a gene dosage-sensitive manner. The critical component behind long fin formation is potassium conductance by the *Kcnj13* channel. Furthermore, we show that this function is conserved in at least four other potassium channels, suggesting that cellular bioelectric activity is the key, not specific potassium channels. These findings reveal a novel function of dermomyotome bioelectricity in zebrafish fin patterning.

## Materials and Methods

All zebrafish were raised and maintained at the Association for Assessment and Accreditation of Laboratory Animal Care (AAALAC)-approved animal housing facilities according to protocols approved by the Purdue Animal Care and Use Committee. The wild-type fish lines used in this study are of the Tübingen/AB (TAB) background. The zebrafish embryos and larvae were sorted and staged according to the staging guides (Kimmel *et al.* 1995; Parichy *et al.* 2009).

### Zebrafish lines

The zebrafish line Dhi2059 was generated by a large-scale retroviral insertional mutagenesis (Golling *et al.* 2002; Amsterdam *et al.* 2004). Stock fish were maintained as described in the zebrafish book (Westerfield 2000). Insertional mutations were identified using inverse PCR as described previously (Allende *et al.* 1996). Genotypes were determined by PCR using gene-specific primers (kcnj13-178F and kcnj13-210R) and virus-specific primer MSL4 (393 bp for the wild-type allele and 228 bp for the mutant allele). Detailed primer sequences are listed in Supplemental Material, Table S1. Transgenic fish line Tg (*col2a1a*: *EGFP-CAAX*) (Dale and Topczewski 2011) and *jaguar* mutant (G157E), *wpr24.e1* were kindly provided by the Washington University zebrafish consortium and David Parichy’s laboratory at the University of Washington, respectively.

### Morphological measurement and skeletal staining

Fin measurements were performed on fish anesthetized with 0.05% tricaine solution. Fish bodies and fin lengths were measured with a caliper. Standard length was measured from snout to caudal peduncle. Linear regression was calculated in Microsoft Excel according to previous reports on fin growth studies (Iovine and Johnson 2000; Perathoner *et al.* 2014). To achieve adequate statistical power, we measured > 20 healthy fish per group/genotype, which depended on fish generated by the genetic crosses. No randomization and blinding was employed.

For adult fish skeletal staining, fish were euthanized with tricaine and fixed in 4% paraformaldehyde for 2 days. The fixed fish were dehydrated with a series of ethanol concentrations (25, 50, 75, and 100%). First, we performed cartilage staining with alcian blue solution (0.01% alcian blue, 70% ethanol, and 30% acetic acid) for 2 days. Fish were treated with a saturated sodium borate solution for 1 day to prevent loss of calcium and followed by 1% KOH treatment for another day. For bone staining, fish were incubated in 1% KOH with alizarin red (0.1%). After destaining with 1% KOH for 2 days, fish were preserved in 100% glycerin via an ascending series of 1% KOH and glycerol (7:3, 5:5, and 3:7).

### Bioinformatics and phylogenetic analyses of the *kcnj13* gene

Exon–intron structure analysis of the *kcnj13* was performed using the University of California, Santa Cruz (UCSC) genome browser, and the gene structure display server (Hu *et al.* 2015) was used for visualization using the information of the *kcnj13* gene downloaded from the table genome annotation in the UCSC zebrafish genome (GRCz10). The *kcnj13* gene complementary DNA (cDNA) information (XM_017350959.1) was retrieved from the National Center for Biotechnology Information, and UTR element analysis was performed using UTRscan with default parameters (Grillo *et al.* 2010). The tertiary structure of KCNJ13 was predicted from the protein sequence in RaptorX (Källberg *et al.* 2012) and visualized in MacPyMOL (v.1.7.6.3).

Inwardly rectifying potassium channel protein sequences were identified by a Basic Local Alignment Search Tool for protein (BLASTp) search using zebrafish Kcnj13 protein sequences as a query in Ensembl. KCNJ13 protein sequences of the representative’s metazoan taxa were selected for alignments and phylogenetic analysis (Tables S2). For each protein, the longest isoform was chosen when there were multiple sequences. Multiple protein sequence alignments were performed in the MUSLE program (Edgar 2004). To identify the best evolutionary model for phylogenetic analysis, a best-model test using maximum likelihood was conducted using default parameters in MEGA6 (Tamura *et al.* 2013). The models with the lowest Bayesian Information Criterion scores were considered to describe the substitution pattern the best, and JTT (Jones-Taylor-Thornton) + G (gamma distribution) (JTT+ G) was chosen. Phylogenetic trees were constructed using the Bayesian analysis method in MrBayes 3.2.6 (Ronquist *et al.* 2012). The following parameters were used for Bayesian phylogenetic analysis: run = 20,000,000, nruns = 2, nchains = 4, aamodel = fixed (Jones), rates = gamma ngammacat = 8, samplefreq = 500, and burninfrac = 0.25.

### cDNA cloning, whole-mount *in situ* hybridization, cryosectioning, and imaging

Total RNAs were isolated from mixed wild-type zebrafish embryos [1–3 d post fertilization (dpf)] using TRIzol reagent (Thermo Scientific) according to the manufacturer’s instructions. Reverse transcriptions were performed using the SuperScript III First-Strand Synthesis System (Thermo Scientific) following the manual’s instructions. Full-length open reading frames (ORFs) of zebrafish *kcnj13* and other genes were amplified by PCR using gene-specific primers (Table S1) designed according to the current protein-coding sequences in Ensembl. The PCR products were then purified using Zymoclean Gel DNA Recovery Kit (Zymo Research) before they were cloned into either the pJet1.2 vector using the CloneJET PCR Cloning Kit (Thermo Scientific) or pDONR221 using BP recombinase. Orientations of gene inserts were verified by Sanger sequencing. Riboprobes were synthesized through *in vitro* transcription using T7 RNA polymerase (New England Biolabs, Beverly, MA) and DIG RNA Labeling Mix (11277073910; Roche). The riboprobes were purified by SigmaSpin postreaction cleanup columns (S5059; Sigma [Sigma Chemical], St. Louis, MO) before use, and stored at −80°. Zebrafish embryos at different developmental stages were collected according to the zebrafish development staging guide (Kimmel *et al.* 1995). Chorions were removed by pronase treatment before fixation. The collected fish embryos were fixed with 4% paraformaldehyde overnight at 4° and then dehydrated using increasing concentrations of methanol (25, 50, 75, and 100%) in PBS with 0.1% Tween-20, and stored at −20°. Whole-mount *in situ* hybridizations were carried out according to previously published methods (Thisse and Thisse 2008; Hensley *et al.* 2016). Color reactions were carried out in alkaline phosphatase buffer (NTMT) solution (100 mM Tris-HCl pH 9.5, 50 mM MgCl_2_, 100 mM NaCl, 0.1% Triton-X, and 1 mM levamisole) with 75 mg/ml nitrotetrazolium blue (NBT) (Roche), 50 mg/ml BCIP (5-bromo-4-chloro-3-indolyl phosphate p-toluidine) (Roche), and 10% DMF (N,N-dimethylformamide). Color development was closely monitored and stopped with NTMT solution when proper color density was achieved. For histological analysis, posthybridization embryos were equilibrated in 15% sucrose, then 30% sucrose in 20% gelatin, after which they were embedded in 20% gelatin for cryosectioning (10–12 µm). Images were acquired using an AxioCam MRc camera on a Zeiss ([Carl Zeiss], Thornwood, NY) Stereo Discovery.V12 and Axio Imager 2 compound microscope.

### Tol2 construct generation and site-directed mutagenesis

The full-length ORFs of zebrafish *kcnj13*, human *KCNJ13* genes, and other zebrafish potassium channel genes (with and without stop codons) were amplified by RT-PCR using gene-specific primers designed according to DNA sequences in Ensembl. PCR products were purified and cloned into the pENTR-D-Topo vector using a pENTR/D-TOPO Cloning Kit (Thermo Scientific) following the manufacturer’s instructions. Site-directed mutagenesis of *kcnj13* was performed using Ex Taq DNA polymerase and an In-Fusion Cloning kit (Takara Bio). Inverse primers that overlapped each other by 15–20 bp at their 5′ ends were designed against the plasmid *actinb (actb2)-kcnj13-EGFP*-pDestTol2pA. Desired mutations were incorporated into the primer pair for each mutant. The primer sequences are listed in Table S1. PCR was performed according to the manufacturer’s instructions, and PCR products were purified using a Zymoclean Gel DNA Recovery kit (Zymo Research) before recircularization at the site of the overlap using a Fusion-Cloning kit. In-Fusion reactions were transformed into Stellar Competent Cells after 15 min incubation at 50°. Each mutant clone was verified by Sanger sequencing before microinjection. The 5.4-kbp *pax3a* promoter was amplified from genomic DNA (gDNA) using primers (Table S1) against the upstream region of the first exon of the *pax3a* gene. The PCR product was inserted into p5E-MCS (Tol2kit #228) linearized by HindIII and BamHI using the In-Fusion Cloning kit (Takara Bio). A three-fragment Gateway cloning-based Tol2 kit was used to generate Tol2 transposon constructs (Kwan *et al.* 2007). Recombination reactions of the *kcnj* genes in pENTR-D-topo and p5E-*actinb*, p3E-*EGFP*pA, p3E-*IRES-EGFP*pA, and pDestTol2pA were performed using LR Clonase II Plus enzyme (Thermo Scientific) following the manufacturer’s instructions. Final clones for each gene were verified by Sanger sequencing.

### CRISPR and T7 endonuclease I assay

The CRISPR RNAs were designed to target the sixth exon of the *kcnj13* gene by the CHOPCHOP program (Montague *et al.* 2014). The DNA sequences of both the RNA template and primers that flank the target region can be found in Table S1. All the guide RNAs (gRNAs) were synthesized by *in vitro* transcription using a HiScribe Sp6 RNA synthesis kit (New England Biolabs) according to the manufacture instruction and published protocol (Gagnon *et al.* 2014). The insertion/deletion (indel) mutations were detected by T7 endonuclease I assay. Briefly, PCRs were performed to amply the target regions, then ∼200 ng of the purified PCR amplicons were denatured at 95° for 10 min and slowly reannealed to facilitate heteroduplex formation (95–85°, −2°/sec and 85°, 1 min; 85–75°, −0.3°/sec and 75°, 1 min; 75–65°, −0.3°/sec and 65°, 1 min; 65–55°, −0.3°/sec and 55°, 1 min; 55–45°, −0.3°/sec and 45°, 1 min; 45–35°, −0.3°/sec and 35°, 1 min; 35–25°, −0.3°/sec and 25°, 1 min; and 12°, hold). Then, the reannealed amplicon was digested with five units of T7 endonuclease I (New England Biolabs) at 37° for 1 hr. Reactions were stopped by adding 1 μl of 0.5 M EDTA, or analyzed by electrophoresis on a 2–2.5% agarose gel and visualized by ethidium bromide staining. For indel mutations, PCR amplicons were cloned into a pJET1.2 vector using the CloneJET PCR Cloning Kit (Thermo Scientific). At least three clones were sequenced for each CRISPR mutant identified by T7 endonuclease I assay.

### Zebrafish transgenesis and microinjection

Tol2 transposase messenger RNA (mRNA) was generated by using the pCS-zT2Tp plasmid (kind gift from Koichi Kawakami) as a template. The plasmid DNA was linearized with Not I and purified using the Zymoclean Gel DNA Recovery kit (Zymo Research). Capped RNA was synthesized using the mMessage mMachine SP6 kit (Thermo Scientific), and poly(A) was added using a Poly(A) Tailing kit (Thermo Scientific). Capped and tailed mRNAs were purified by lithium chloride precipitation and dissolved in DEPC-treated water. Tol2 expression constructs (25 ng/µl) and Tol2 mRNA (25 ng/µl) with 0.025% phenol red (P0290; Sigma) were injected into one-cell-stage zebrafish embryos under a dissection microscope (SMZ445; Nikon, Garden City, NY) using a PV820 pneumatic PicoPump (World Precision Instruments). Next 8–10 injected embryos were sampled for gDNA isolation using the HotSHOT method (Truett *et al.* 2000). An excise assay was performed to verify Tol2 transposon efficiency (Kawakami and Shima 1999). The rest of the injected fish embryos were raised to adulthood once the excise assays confirmed the Tol2 activity. F_0_-injected adult fish were crossed to wild-type fish, and positive transgenic zebrafish embryos with fluorescence were sorted and raised to adulthood as stable F_1_ transgenic fish lines.

### Cell culture and transfection

We chose the HEK293T cell line, due to its easy transfection and frequent use is electrophysiology studies. It was acquired from the American Type Culture Collection and maintained in the laboratory. About 2 × 10^5^ cells were seeded in a six-well plate and cultured for 24 hr in a humidified 37° incubator with 5% CO_2_. Dulbecco’s Modified Eagle Medium with 10% fetal bovine serum was changed on the second day. For each well, 2.0 μg of plasmid DNA and 7.5 μl Lipofectamine 2000 were mixed in 100 μl of Opti-MEM I medium without serum. After incubation for 5 min at room temperature, the solution was added dropwise to the cultured cells. Cells were cultured for 24 hr and were subjected to either patch-clamp or luciferase assay.

### Patch-clamp electrophysiology

Whole-cell patch-clamp experiments were conducted in normal Tyrode’s solution (115 mM NaCl, 20 mM KCl, 1.8 mM CaCl_2_, 0.53 mM MgCl_2_, 5.5 mM D-glucose, and 5.5 mM HEPES). Pipette internal solution (5 mM KCl, 130 mM K-gluconate, 10 mM HEPES, 2 mM MgATP, 0.3 mM Na_2_GTP, and 0.6 mM EGTA with pH adjusted to 7.3 and osmolarity adjusted to 285 mOsm) was used. Patch-clamp electrodes were pulled from filamented borosilicate glass tubes (BF150-86-10; Sutter Instruments) using a P-97 micropipette puller (Sutter Instruments) to a resistance of 3.5–7.9 MΩ. Data were acquired through Multiclamp 700B and digitized by Digidata 1550 (Molecular Devices). Cells were held at −80 mV for voltage clamp recordings. A total of 20 steps ranging from −140 to +50 mV were recorded under voltage clamp at 10-mV steps. Membrane capacitance was measured by Membrane Test (Molecular Devices). Resting potentials were extracted from the voltage–current curve for each cell where current equaled 0 nA. Raw traces generated by Clampex software were analyzed using custom-written scripts in Python. Mutant constructs were measured blindly. Any data points that fell outside of three interquartile ranges (IQRs) were defined as outliers and excluded from analysis. There was no randomization procedure.

### Luciferase assay

Zebrafish exon 5 and corresponding cDNAs were cloned into the pMuLE ENTR SV40 Luc2 (FF-Luciferase) L5-L2 vector using Fusion cloning. The multiple lentiviral expression (MuLE) system kit was a gift from Ian Frew (Addgene kit #1000000060) (Albers *et al.* 2015). The primers used are listed in Table S1. *Renilla* luciferase (pRL-TK) was used as a reference. After transient transfection into HEK293T cells as described above, luciferase activities were measured using the Dual-Luciferase Reporter Assay System (Promega, Madison, WI). For each construct, experiments were repeated at least twice in triplicate. Unpaired Student’s *t*-tests and one-way ANOVA were chosen for statistical significance evaluations.

### Data availability

Reagents are available upon request. The authors affirm that all data necessary for confirming the conclusions of the article are present within the article, figures, and tables. Supplemental material available at figshare: https://doi.org/10.25386/genetics.12445469.

## Results

### Paired and median fins are elongated in the zebrafish insertional mutant Dhi2059

**Figure 1:**
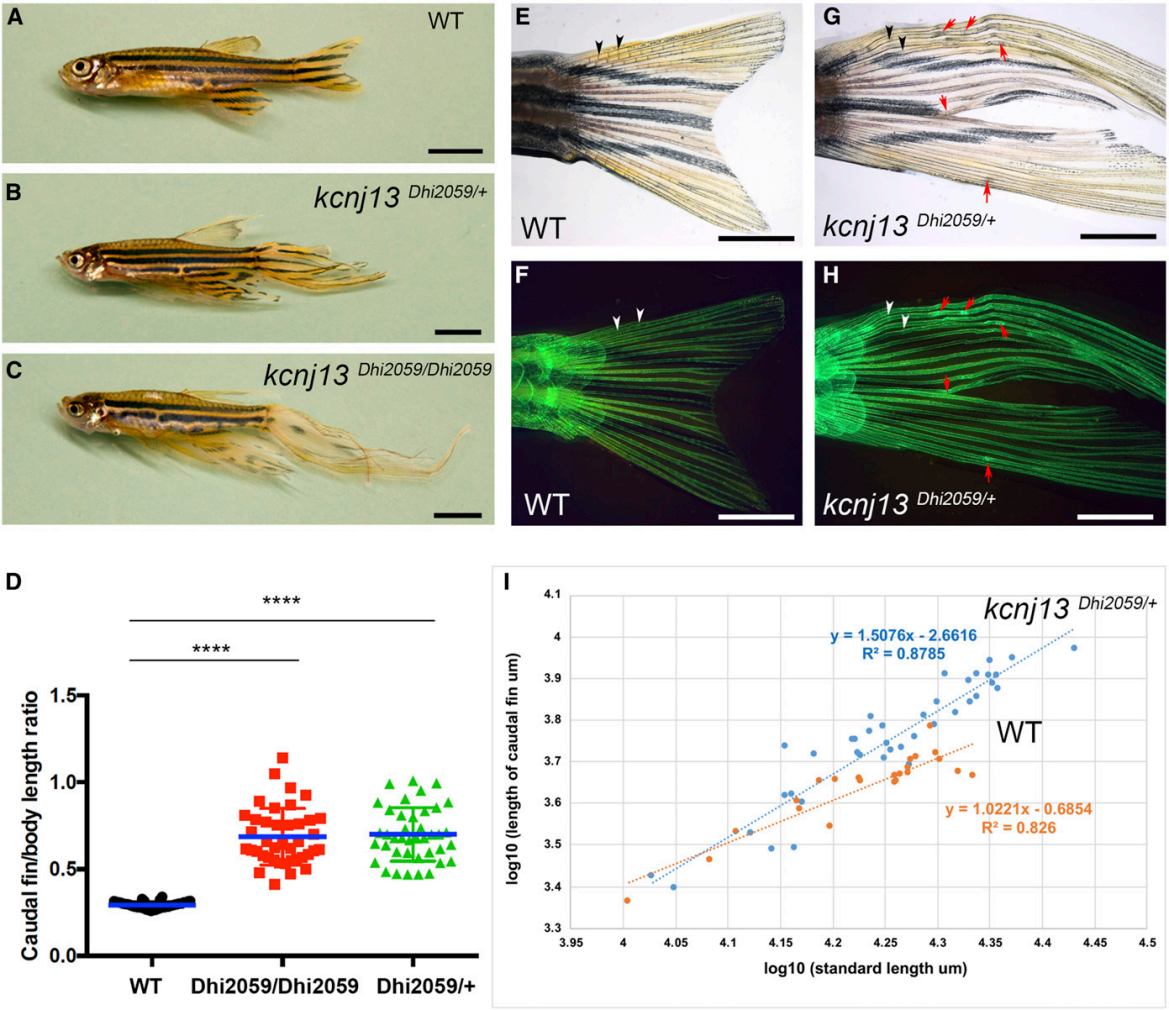
Fins are elongated in the adult zebrafish mutant Dhi2059. (A) WT fish with normal-sized fins. (B and C) Both one- and two-copy transallelic mutants of Dhi2059 possessed elongated paired and median fins. Bars, 50 mm in (A–C). (D) The lengths of caudal fins in Dhi2059 mutants are significantly longer compared to WT siblings. Fish were generated by *kcnj13^Dhi2059/+^* incrossing. For each group, *n* = 40 (20 males and 20 females). * *P* < 0.0001 by ordinary one-way ANOVA test (Tukey’s multiple comparisons) based on mean difference and SD. (E and F) Normal fin ray segments in WT fish. (G and H) In the Dhi2059 mutant, the fin ray segments of the caudal fins are increased. The number of fin ray joints are barely noticeable. Black/white arrowheads indicate natural ray joints. Red arrows indicate the healing scars of broken bone structures. (E–H) Tg (*col2a1a*: EGFP-CXXC) labeled WT and Dhi2059 mutant fish. Bars, 25 mm in (E–H). (I) Allometric growth of caudal fins in *kcnj13^Dhi2059/+^* mutants (*n* = 39; *k* = 1.5) compared to isometric WT siblings (*n* = 23, *k* = 1.0), reflected by linear regression analysis on the lengths of caudal fins and standard body lengths (measured from the tips of the snouts to the posterior ends of the last vertebrae). *k* = allometric coefficient. The slopes are significantly different, *P* = 0.002, F-test of general linear model analysis. All fish were generated by incrossing of *kcnj13^Dhi2059/+^* fish. WT, wild-type.

The zebrafish mutant Dhi2059 arose from a large-scale Moloney murine leukemia virus (MMLV) retroviral insertional mutagenesis screen (Golling *et al.* 2002; Amsterdam *et al.* 2004), and developed elongated fins compared to wild-type siblings (Figure 1, A–C). This long-finned phenotype is inherited dominantly. Two-copy transallelic fish (*kcnj13^Dhi2059/Dhi2059^*) are viable and fertile, and their fin lengths are similar to those of the *kcnj13^Dhi2059/+^*, as determined by the lengths of caudal fins (Figure 1D). The only noticeable difference between the one-copy and two-copy transallelic adult fish (*kcnj13^Dhi2059/Dhi2059^* and *kcnj13^Dhi2059/+^*) is the interrupted second pigmentation stripe (Figure 1, B and C). Elongation of the fins in the Dhi2059 mutants is due to increased lengths of lepidotrichia (Figure 1, E–H); proximal (radial) skeletal elements remain unchanged (Figure S1). Fish barbel lengths remained unchanged (Figure S2). Segmentation of lepidotrichia is dramatically decreased, and the ray joints are barely noticeable in the mutant (Figure 1, G and H). These long lepidotrichia usually break when the fish swim, leading to skeletal fractures/scars (Figure 1, G and H). Fin elongation can be noticed as early as 1.5 months after fertilization, suggesting that fin growth is allometric (disproportionate growth rate in different portions of an organism) in the Dhi2059 mutants (Iovine and Johnson 2000). Using the caudal fin as an example, we measured the lengths of the bodies and caudal fins of 7–8-week-old fish and performed linear regression analysis. The wild-type fish caudal fins (*vs.* standard body length) showed isometric growth (proportionate growth) patterns (*k* = 1.0), while the Dhi2059 mutants followed an allometric growth pattern (*k* = 1.5) (Figure 1I). This allometric growth factor is similar to two other long-finned mutants, *kcc4a* (*k* = 1.35) and *kcnk5b* (=1.88) (Perathoner *et al.* 2014; Lanni *et al.* 2019), and all these mutants share elongated lepidotrichia.

### The *kcnj13* gene is disrupted in the Dhi2059 mutant

**Figure 2:**
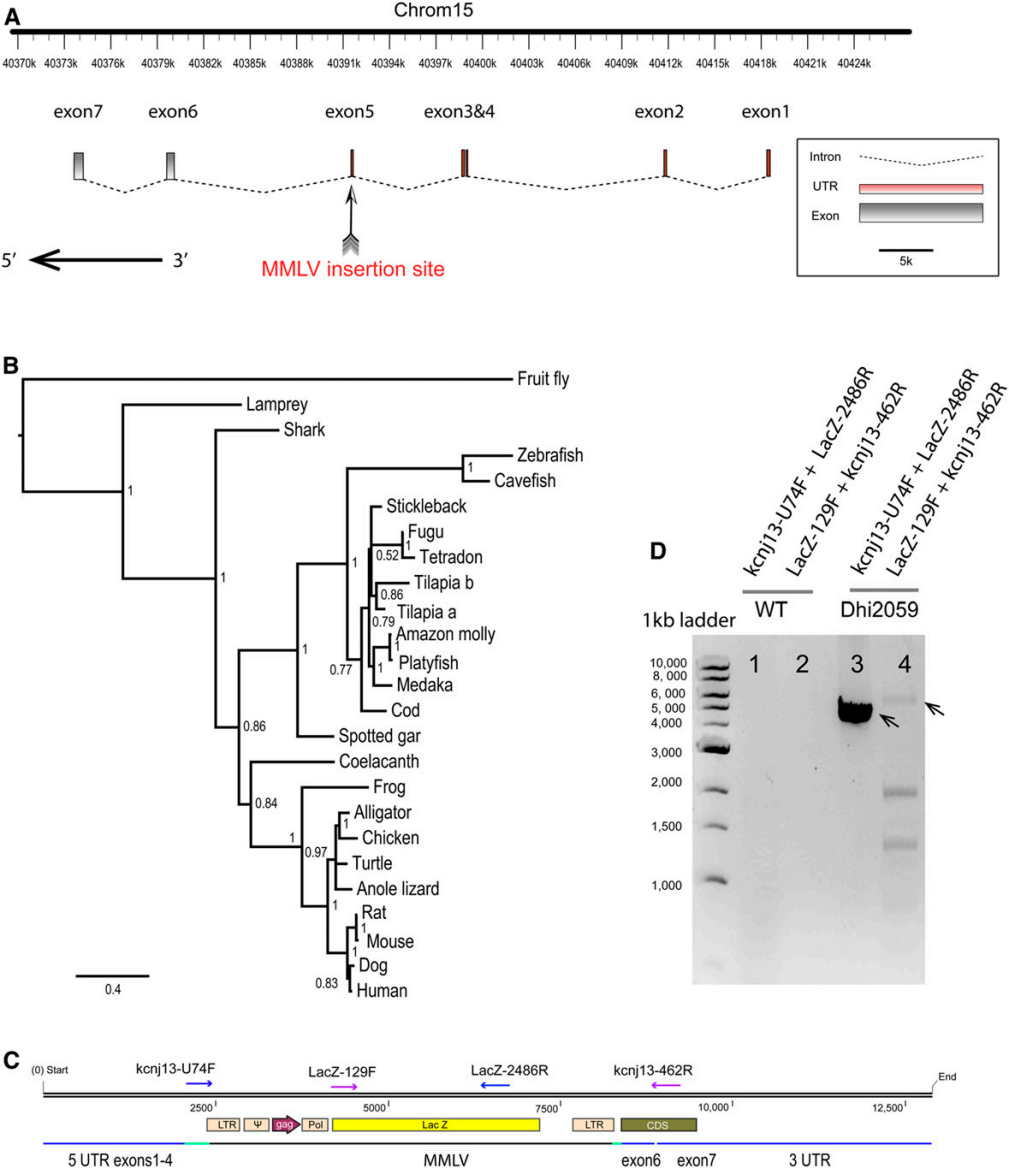
Viral insertion was identified in the *kcnj13* gene locus and mRNA. (A) Schematic of the zebrafish *kcnj13* gene denoting the position of viral insertions in the hi2059 mutant. The viral DNA randomly inserted into the fifth exon of the gene. The horizontal black arrow indicates the protein-coding area. (B) Extended majority-rule consensus phylogenetic tree for the Bayesian analysis of KCNJ13 proteins. Numbers at each node indicate pp values based on 20 million replicates. Branch lengths are proportional to means of the pp densities for their expected replacements per site. (C) The diagram of the viral insertion in the *kcnj13* mRNA. The approximate positions of PCR primers are indicated with purple/blue arrows. Green lines indicate broken exon 5, and blue lines indicate the remaining exons. (D) Confirmation of chimera viral sequence and *kcnj13* mRNA by RT-PCR. The expected sizes of PCR products are 5708 bp (Lacz-129F + kcnj13-462R) and 4509 bp (kcnj13-U74F + LacZ-2486R), respectively. The smaller bands in lane 4 are nonspecific amplified bands. kcnj13-U74F and kcnj13-462R are targeted at the beginning of exons 5 and 7, respectively. CDS, coding DNA sequence; LTR, long terminal repeat; mRNA, messenger RNA; pp, posterior probability; UTR, untranslated region.

To identify the genetic alteration responsible for this mutant, we mapped the Dhi2059 viral insertion in the fifth exon of the *kcnj13* gene (Figure 2A) using inverse PCR (Allende *et al.* 1996). The *kcnj13* gene, also known as *kir7.1*, encodes an inwardly rectifying potassium channel, which is an evolutionarily conserved gene in metazoans (Figure 2B). A BLASTp search followed by phylogenetic analyses identified a single *kcnj13* gene in the zebrafish genome. The *kcnj13* gene has seven exons in total, but only the last two exons code for the protein (Figure 2A). Since the viral DNA was inserted into the fifth exon, which is ∼11 kb upstream of the protein-coding region, the protein-coding sequence was most likely not affected. Instead, the insertion may interrupt the function and/or stability of the mRNA, or influence the temporal and spatial expression of the gene at the transcriptional and translational levels. Thus, we searched for the presence of viral sequences in the mRNA by RT-PCR (Figure 2C). We were able to amplify PCR products of the expected size using retrovirus- and *kcnj13*-specific primers (Figure 2D), confirming that viral sequences were transcribed and thus detected as a chimeric cDNA. Genetic linkage analysis revealed that the insertion is 100% linked with the long-finned phenotype. Thus, the *kcnj13* gene coding DNA and RNA are disrupted in the Dhi2059 mutant.

The wild-type *kcnj13* cDNA has a long 5′ UTR, which likely contains regulatory elements such as an upstream open reading frame (uORF) or internal ribosome entry site (IRES). Therefore, we performed a UTR element analysis for the *kcnj13* cDNA and identified 11 uORFs by UTRScan (Figure S3). Since the viral insertion disrupted the first uORF, and uORFs usually inhibit protein translation (Calvo *et al.* 2009; Barbosa *et al.* 2013), we hypothesized that the viral insertion might block the inhibition of Kcnj13 protein translation by interrupting the first uORF. To test this hypothesis, we cloned the first uORF and inserted it into an SV40 promoter-driven firefly luciferase construct and then performed luciferase assays in HEK293T cells. Indeed, the first uORF was able to reduce the firefly luciferase expression. Furthermore, the firefly luciferase expression could be partially rescued by a 329-bp long terminal repeat (LTR) sequence from MMLV (Figure S3). Thus, the retroviral DNA insertion may release the translational inhibition of the uORF, and the translation of kcnj13 does not dramatically decrease, despite the integration of the retrovirus into the *kcnj13* mRNA.

### *Kcnj13* is ectopically and transiently expressed in the somite and dermomyotome during zebrafish development

**Figure 3:**
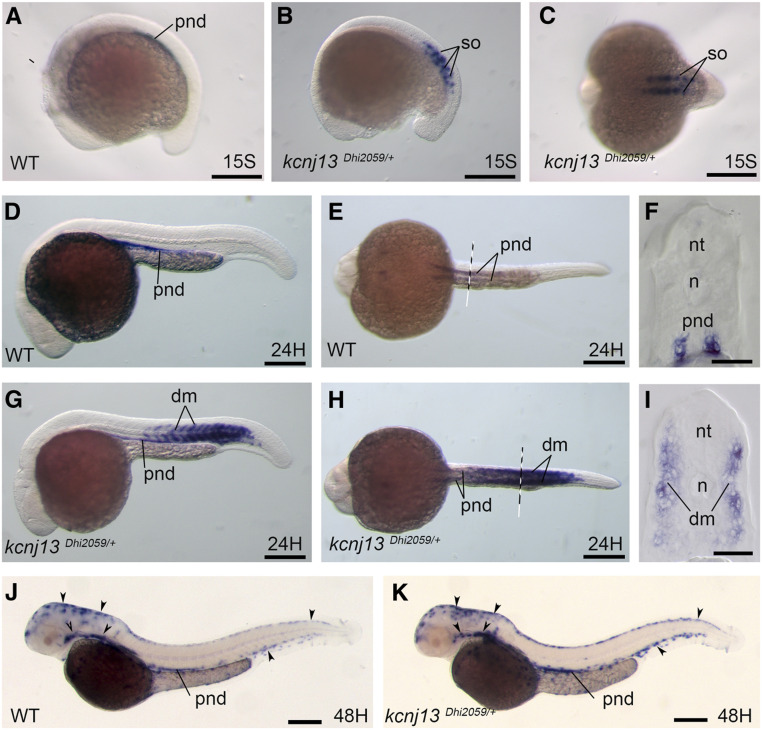
*Kcnj13* is ectopically expressed in somites of the Dhi2059 mutant. Whole-mount *in situ* hybridizations of zebrafish embryos at the stages from 15S to 48 hpf were performed. WT embryos (A, D–F, and J). *kcnj13^Dhi2059/+^* fish embryos (B, C, G–I, and K). (A) *Kcnj13* is expressed at the pnd in WT embryos. (B) In the *kcnj13^Dhi2059/+^* mutant, *kcnj13* is expressed in the somites at the 15S stage. (C) Dorsal view of the embryo in (B). (D–F) At the 24-hpf stage, *kcnj13* is continually expressed in the pronephric ducts of WT embryos. (D) Lateral view. (E) Ventral view of the 24-hpf embryos. The dashed line indicates the approximate position of the transverse section in (F). (G–I) The expression of *kcnj13* in the *kcnj13^Dhi2059/+^* mutant at the 24-hpf stage. The *kcnj13* mRNA is also expressed in the somites in addition to pnds. (H) Ventral view of the same embryo in (G). The dashed line indicates the position of the transverse section in (I). (I) Transverse section revealed that *kcnj13* is expressed in the dm. (J) At the 48-hpf stage, *kcnj13* is continually expressed in pnds in WT fish embryos, and it is also expressed in melanocytes (arrowheads). (K) In the *kcnj13^Dhi2059/+^* fish embryos, *kcnj13* expression is similar to WT embryos at 48 hpf. The somite expression is no longer detectable. Bar, 250 μm (A–E, G, H, J, and K). Bar, 100 μm (F and I). 15S, 15-somite; dm, dermomyotome; hpf, hours postfertilization; mRNA, messenger RNA; n, notochord; nt, neural tube; pnd, pronephric ducts; so, somite, WT, wild-type.

To test whether the viral insertion impacts *kcnj13* gene transcription in the Dhi2059 mutant, we examined *kcnj13* mRNA expression during zebrafish embryonic development using whole-mount *in situ* hybridization. *Kcnj13* is usually expressed in the pronephric duct from the 15-somite stage to 48 hpf (hours postfertilization) and in the melanocyte cells at 48 hpf (Figure 3, A, D–F, and J). Contrary to the wild-type fish, *kcnj13* was ectopically expressed in the somites at the 15-somite stage in Dhi2059 mutant embryos (Figure 3, B and C). At 24 hpf, in addition to the pronephric duct, *kcnj13* expression was evident in a somite derivative, the dermomyotome (Figure 3, G–I). This dermomyotome expression domain remained at 36 hpf (data not shown), but was undetectable by 48 hpf (Figure 3K). This transient ectopic expression of *kcnj13* in somites and the dermomyotome suggests that the region (around the fifth exon) of viral DNA insertion may contain a tissue- and temporal-specific silencer. At these developmental stages, *kcnj13* was not found in the fin folds, embryonic structures from which the dermoskeleton develops, except in the pigmentation cells around the bases of the fin buds. In addition, *kcnj13* expression in the pigment cells appeared unchanged between the Dhi2059 mutant and wild-type fish embryos (Figure 3, J and K). Moreover, we examined *kcnj13* gene expression in the fin buds/fin anlagen up to 2 weeks after fertilization, and no *kcnj13* was detected (Figure S4).

The signaling centers critical for normal appendage development are highly conserved in fish fins and tetrapod limbs (Iovine 2007). The apical ectodermal ridge/fold (AER/AEF), for example, has been detected in both fins and limbs and regulates proximal–distal outgrowth. The zone of polarizing activity (ZPA) determines the anterior–posterior axis in both types of appendages. To assess whether these signaling pathways were affected in the Dhi2059 mutant, we examined gene markers (*fgf8a*, *shha*, and *hoxd13a*) associated with their activity, as well as markers of fin skeletogenesis (*and1*, *col2a1a*, and *sox9a*). We did not find any noticeable differences in expression levels of these marker genes in the 3 dpf mutants and wild-type embryos (Figure S5).

### Allele-specific kcnj13 loss-of-function mutant rescues fin elongation in the Dhi2059 mutant

**Figure 4:**
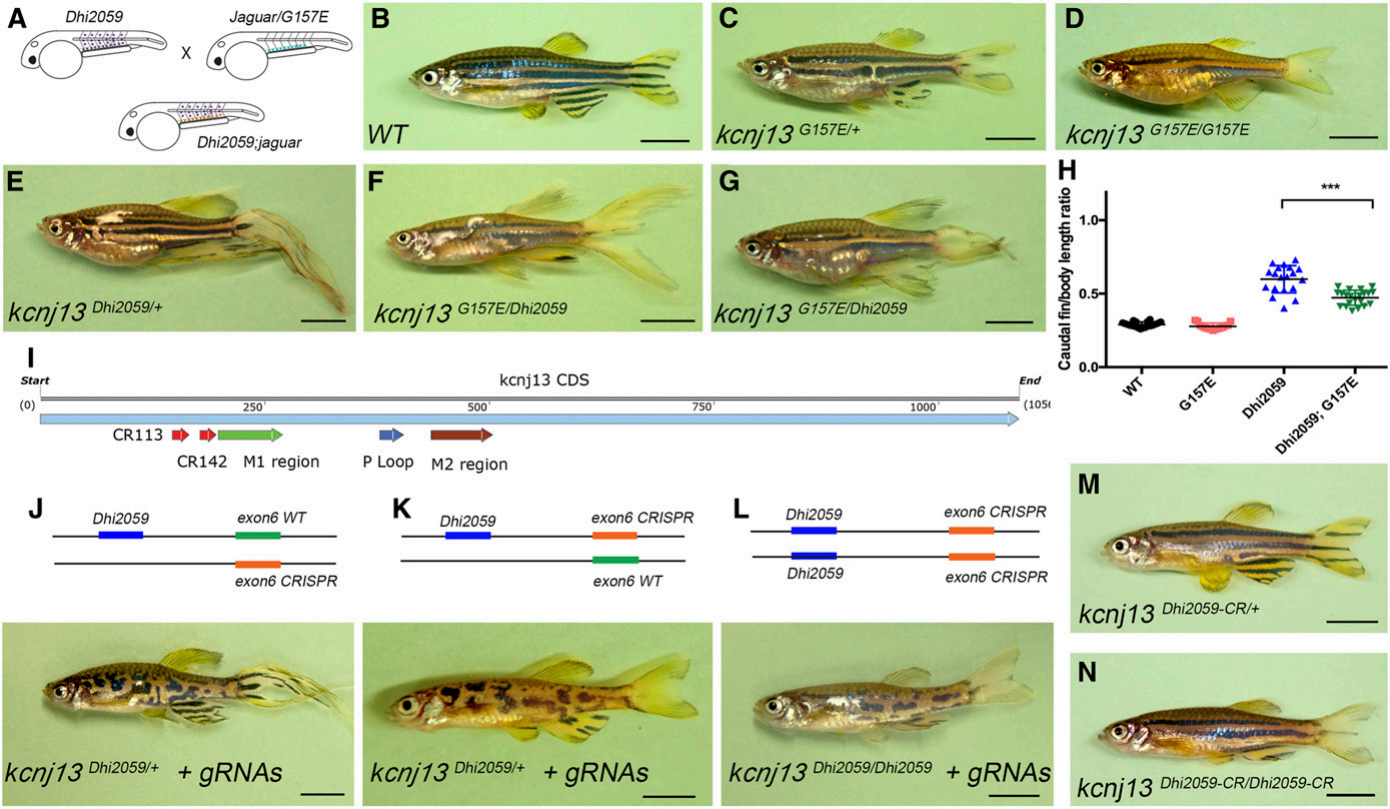
Dhi2059 long-finned phenotype was only able to be rescued by a *kcnj13* loss-of-function mutation in an allelic-specific manner. (A) Illustration for principle of the genetic rescue experiment. Purple dots represent WT *kcnj13* gene expression in both endogenous domains and ectopic expression in somites. The blue dots represent the loss-of-function *kcnj13* endogenous expression by the *jaguar* (Kcnj13^G157E/^) allele, which is driven by an intact promoter. The orange dots represent the overlapped expression. The green lines indicate pronephric ducts. (B) WT control fish. (C) Morphology of heterozygous *jaguar* mutant. (D) Morphology of homozygous *jaguar* mutant. (E) Morphology of *kcnj13^Dhi2059/+^* mutant. (F and G) Morphology of double heterozygous *jaguar* and Dhi2059 mutants, *kcnj13^Dhi2059/G157E^*. (F) Male. (G) Female. Note, the caudal fin broke due to its large size before imaging. Although pigmentation patterns were altered, the long-finned phenotype still remained in *kcnj13^Dhi2059/G157E^* mutants. (H) Quantitative comparison between *kcnj13^Dhi2059/+^* mutant and *kcnj13^Dhi2059/G157E^* mutants. WT (*n* = 20); G157E, *jaguar* (G157E, *n* = 15); Dhi2059 (*n* = 19); Dhi2059; G157E (*n* = 21). * *P* < 0.0001 by unpaired Student’s *t*-test. (I) Location of CRISPRs against *kcnj13* coding region. CRISPR and important functional domains are annotated with colored arrows. (J–L) illustration and morphology of CRISPR-induced insertion/deletion mutation in Dhi2019 mutants. All fish shown are injected F_0_ adults. (J) CRISPR mutation is located on the WT allele (orange bar) of *kcnj13^Dhi2059/+^*. (K) CRISPR mutation is located on the virial inserted allele (orange bar) of *kcnj13^Dhi2059/+^*. (L) CRISPR mutations are located on both virial inserted alleles (orange bars) of *kcnj13^Dhi2059/Dhi2059^*. (M–N) F3 generation adult fish with CRISPR mutant that linked with Dhi2059 inserted *kcnj13* allele. (M) Morphology of heterozygous *kcnj13* ^hi2059-CR^mutant. (N) Morphology of homozygous *kcnj13*^hi2059-CR/hi2059-CR^ mutant. Long fins are completely rescued in both conditions. Bars, 5 mm. CR, CRISPR mutant; CRISPR, clustered regularly interspaced short palindromic repeats; gRNA, guide RNA; WT, wild-type.

Since Kir channels work as a tetramer (Yang *et al.* 1995), the overall channel conductance may be reduced if the tetramers are composed of wild-type and mutant proteins due to a stoichiometric effect. We reasoned that the *kcnj13* loss-of-function mutant can disrupt/reduce long fin formation if it is coexpressed with *kcnj13* from Dhi2059 in the same cells (Figure 4A). To test this hypothesis, we crossed a *kcnj13* loss-of-function homozygous mutant, *jaguar* (*kcnj13^G157E^*), which was found to have disrupted pigmentation patterning (Figure 4, B–D) (Iwashita *et al.* 2006), and our Dhi2059 mutant (Figure 4E) and examined the fin sizes of the adults. To our surprise, the transallelic mutants (*kcnj13*^G157E/Dhi2059^) still develop long fins (Figure 4, F and G). However, the ratio of caudal fin relative to standard body length was reduced quantitatively (Figure 4H). Interestingly, *kcnj13^Dhi2059/G157E^* fish possess a pigmentation phenotype (broken second horizontal strip) that is similar to that of the *jaguar* homozygotes, *kcnj13^G157E/G157E^*. The *jaguar-kcnj13* allele is regulated by the wild-type promoter and is only expressed in physiologically active tissues (such as the pronephric duct and pigment cells), and is not aligned with the Dhi2059-*kcnj1*3 allele in the dermomyotome ectopic domain (Figure 4A). Overall, these results suggest that the dermomyotome ectopic domain is mainly responsible for the elongated fins.

To further demonstrate that ectopic dermomyotome expression of the Dhi2059-*kcnj1*3 allele is the key for long fin development, we reasoned that mutation of the *kcnj1*3 allele with the Dhi2059 viral insertion would be able to restore normal fin development. Crossing Dhi2059 with the *jaguar* (G157E) cannot generate a compound mutant with both viral DNA insertions (Dhi2059) in the same genetic locus because of the short distance between the two genetic changes. Therefore, we turned to the CRISPR system to generate Dhi2059 allele-specific kcnj13 mutants. We designed two gRNAs against *kcnj13* exon 6, a region that is 160–200 bp downstream of the ATG start codon (Figure 4I), and injected gRNAs into Dhi2059 mutant fish embryos. The injected F_0_ fish were raised to adulthood, genotyped for both Dhi2059 and CRISPR mutations, and were screened for long *vs.* normal fins. We identified fish carrying both Dhi2059 and CRISPR mutations, but that had normal-sized fins. We then sequenced a few CRISPR mutants with and without long fins, outcrossed them with wild-type fish to generate F_1_ generation adults, and examined the linkage of these two mutations. DNA sequencing validated truncated mutants created by CRISPR (Figure S6). The normal-fin fish with both Dhi2059 and CRISPR mutations were indeed linked in the F_1_ adult fish, but the two mutants were not linked in long-finned fish (genotype data not shown). Elongated fins persisted in the fish with the CRISPR mutation on the *kcnj13* isoallele (Figure 4J), but not in fish with linked Dhi2059 and CRISPR mutations in both *kcnj13^Dhi2059/+^* and *kcnj13^Dhi2059/Dhi2059^* mutants (Figure 4, K and L). We also outcrossed the linked CRISPR mutant (*kcnj13^Dhi2059-CR/+^*) with wild-type fish to the F_3_ generation. Both heterozygous and homozygous mutants rescued the elongated fins with 100% penetration (Figure 4, M and N). Thus, these data further support the idea that ectopic dermomyotome expression of *kcnj13* is mainly responsible for the subsequent appearance of elongated fins.

### Transient ectopic expression of *kcnj13* in the dermomyotome phenocopies the long-finned phenotype

**Figure 5:**
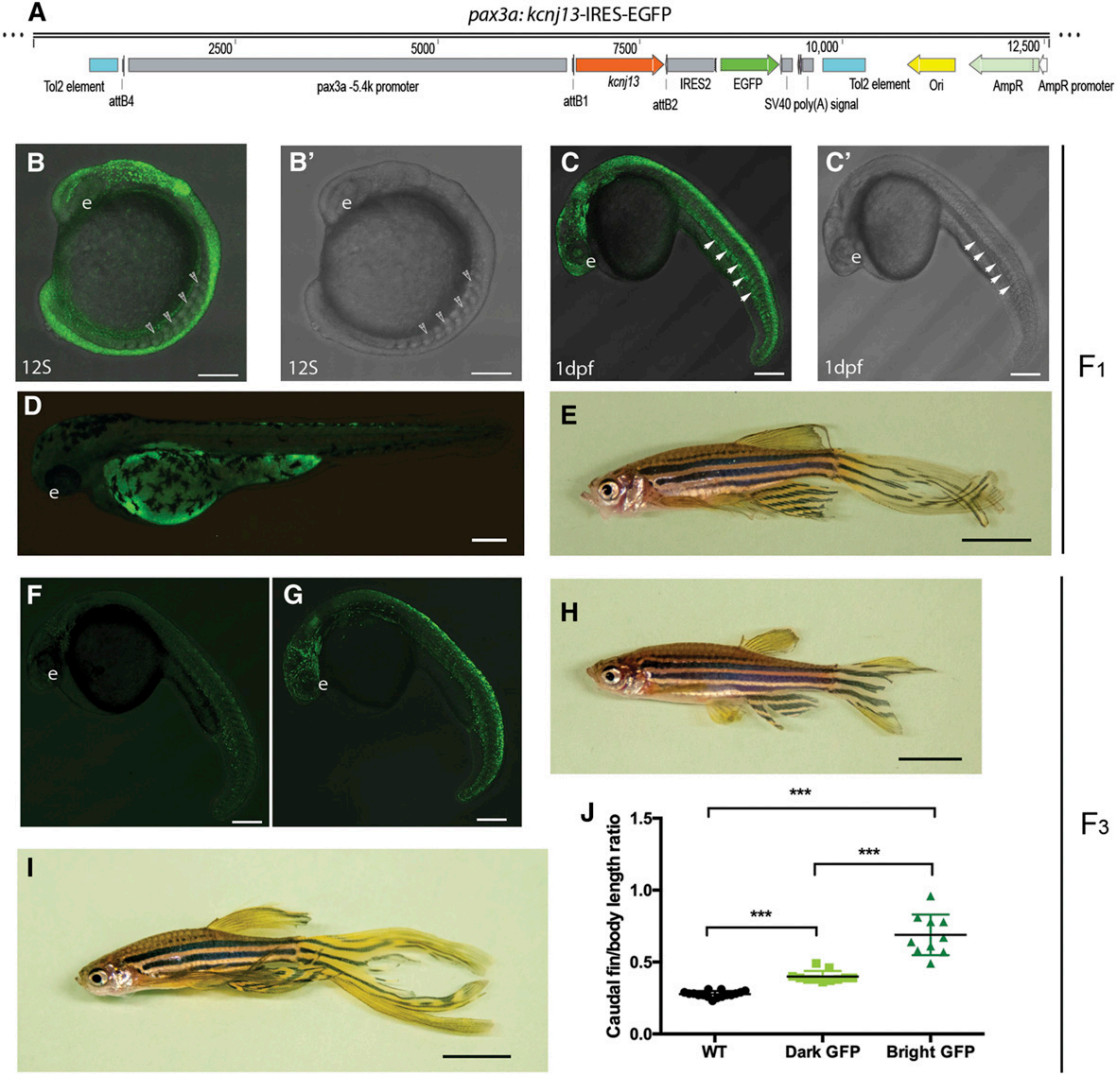
Transient ectopic expression of *kcnj13* by *pax3a* promoter phenocopies Dhi2059 elongated fins. (A) Schematic illustration of a construct (*pax3a:kcnj13*-IRES-EGFP) used for Tol2 transgenesis. A 5.4-kbp-long *pax3a* promoter drives *kcnj13* and independent EGFP expression. (B–E) Representatives of F_1_ generation of *pax3a:kcnj13*-IRES-EGFP transgenic fish. (B) EGFP expressed in the somites of a 12S-stage fish embryo. White arrowheads indicate the somites. (B’) Bright-field image of the same embryos in (B). (C) EGFP expressed in the somites and dermomyotome of a 24-hpf-stage fish embryo. White arrows indicated the dermomyotome. (C’) Bright-field image of the same embryos in (C). (D) EGFP expression disappears from somites in a 48-hpf-stage zebrafish embryo. Only autofluorescence is visible. (E) Representative gross morphology of an adult Tg(*pax3a:kcnj13*-IRES-EGFP). Elongation of the fins is similar to the Dhi2059 mutant. (F–I) Representatives of F_3_ generation of *pax3a:kcnj13*-IRES-EGFP transgenic fish. (F). A dark-EGFP fish embryo. (G) A bright-EGFP fish embryo. (H) An adult dark-EGFP transgenic fish. The fin is slightly elongated compared to WT. (I) An adult bright-EGFP transgenic fish. (J) Comparison of the ratio of caudal fin over standard body length among no-EGFP (*n* = 16), dark-EGFP (*n* = 11), and bright-EGFP (*n* = 10) adult transgenic fish. *) *P* < 0.0001 by pairwise Student’s *t*-test. Bars, 200 μm (B–D, F, and G), Bars, 5 mm (E, H, and I). 12S, 12-somite; e, eye; EGFP, enhanced GFP; IRES, internal ribosome entry site; WT, wild-type.

Long-term cell lineage tracking has demonstrated that the dermomyotome of the somite gives rise to the dermal skeleton in zebrafish and medaka, including the fin rays and scales (Lee *et al.* 2013a,b; Shimada *et al.* 2013). Moreover, somites not only participate in median fin development (Freitas *et al.* 2006), but have also recently been revealed to contribute to the AER/AEF in zebrafish (Masselink *et al.* 2016). Thus, we reasoned that transient ectopic expression of *kcnj13* in the dermomyotomes of somites is the key to elongated fin development. To further test this hypothesis, we cloned a 5.4 kbp promoter of *pax3a*, which is transiently expressed in the dermomyotome (Feng *et al.* 2006; Hammond *et al.* 2007). Then, we created a transgenic fish line, Tg (*pax3a*-5.4k: *kcnj13-IRES-EGFP*), to express *kcnj13* in the dermomyotome and imitate Dhi2059 *kcnj13* ectopic expression (Figure 5A). Enhanced GFP (EGFP) confirmed expression in the dermomyotome from the 12-somite to 24-hpf stages (Figure 5, B–D) and F_1_ transgenic fish developed long fins, thereby phenocopying the Dhi2059 mutant (Figure 5E). To examine whether the *kcnj13* gene copy number was related to fin length, we established a transgenic fish line and outcrossed it with wild-type fish to the F_2_ generation. Then, we incrossed these F_2_ generation fish, and sorted them out roughly by EGFP intensity into dark-GFP and bright-GFP groups under a fluorescent microscope. Once they grew to adults, we examined their fins by measuring caudal fin-standard body lengths. Indeed, the bright-GFP fish developed longer fins (Figure 5, F–J), suggesting that dermomyotome bioelectricity exerts a gene dosage effect on fin pattering.

### The long-finned phenotype is dependent on Kcnj13 potassium channel conductance

**Figure 6:**
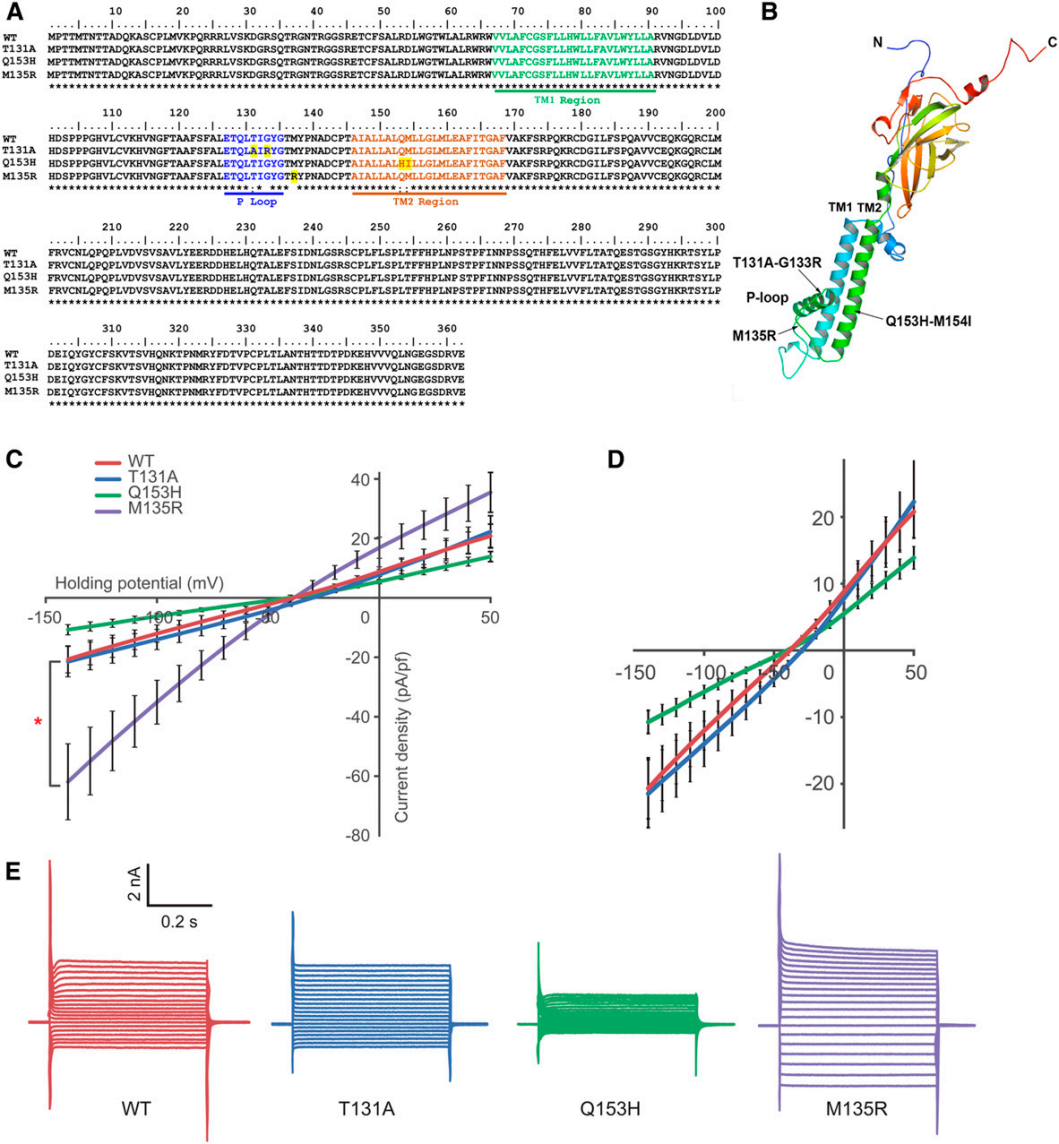
Generation of Kcnj13 mutants with different potassium conductance. (A) Protein sequence alignment of site-directed mutants. TM1, TM2, and P-loop domains are highlighted in green, brown, and blue, respectively. Altered amino acids are highlighted in yellow. (B) A computation-predicted three-dimensional structure of the Kcnj13 subunit. Mutated sites are indicated with arrows. (C) Conductance characterization of three Kcnj13 mutants compared to WT showing decreased conductance of Q153H (*n* = 12, *P* = 0.022) and increased conductance of M135R (*n* = 19, *P* = 0.020). The mutant T131A did not show a significant difference in conductance (*n* = 12, *P* > 0.9) compared to WT (*n* = 13). The current density was calculated by dividing current (pA) at each holding potential by the cell membrane capacitance (pF) to normalize the difference in the cell size. Statistical differences of mean values were calculated using a two-tailed Student’s *t*-test. (D) Replotted part of (C) with enlarged *y*-axis to show the difference between the T131A mutant and the WT in resting membrane potential (also see Figure S7). (E) Example recording traces from each mutant and WT channels. Holding potential was −80 mV and step size was 10 mV. P-loop, pore-forming; TM, transmembrane; WT, wild-type.

Kcnj13 is a Kir channel that is usually a contributor to the cell membrane potential and cell bioelectricity (Hibino *et al.* 2010). Therefore, we next asked whether the potassium conductance of this channel is essential for the formation of long fins. To address this question, we first performed site-directed mutagenesis of the functional domains (TM1, TM2, and P-loop) of the Kcnj13 protein (Figure 6, A and B). Two loss-of-function mutants (T131A and Q153H) and one gain-of-function mutant (M135R), which is equivalent to human KCNJ13 M125R (Krapivinsky *et al.* 1998), were created and inserted into Tol2 transposon vectors using a zebrafish ubiquitous expression promoter, *actinb* (Kwan *et al.* 2007). We chose this promoter because it allowed us to perform subsequent experiments in both human cells and zebrafish. Then, we transiently transformed HEK293T cells, and positively transformed cells were selected by green fluorescence. Corresponding conductivities of these three zebrafish mutant Kcnj13 channels in HEK293T cells were verified using patch-clamp electrophysiology. Both T131A and Q153H were indeed less conductive, and the M135R mutant was more conductive (Figure 6, C–E). Interestingly, electrophysiological traces of the T131A mutant were similar to wild-type Kcnj13 (Figure 6, C and D), while the resting membrane potentials of the transfected HEK293T cells were more depolarized compared to other mutant and wild-type Kcnj13 channels (Figure S7), suggesting that T131A altered its conductance property.

**Figure 7:**
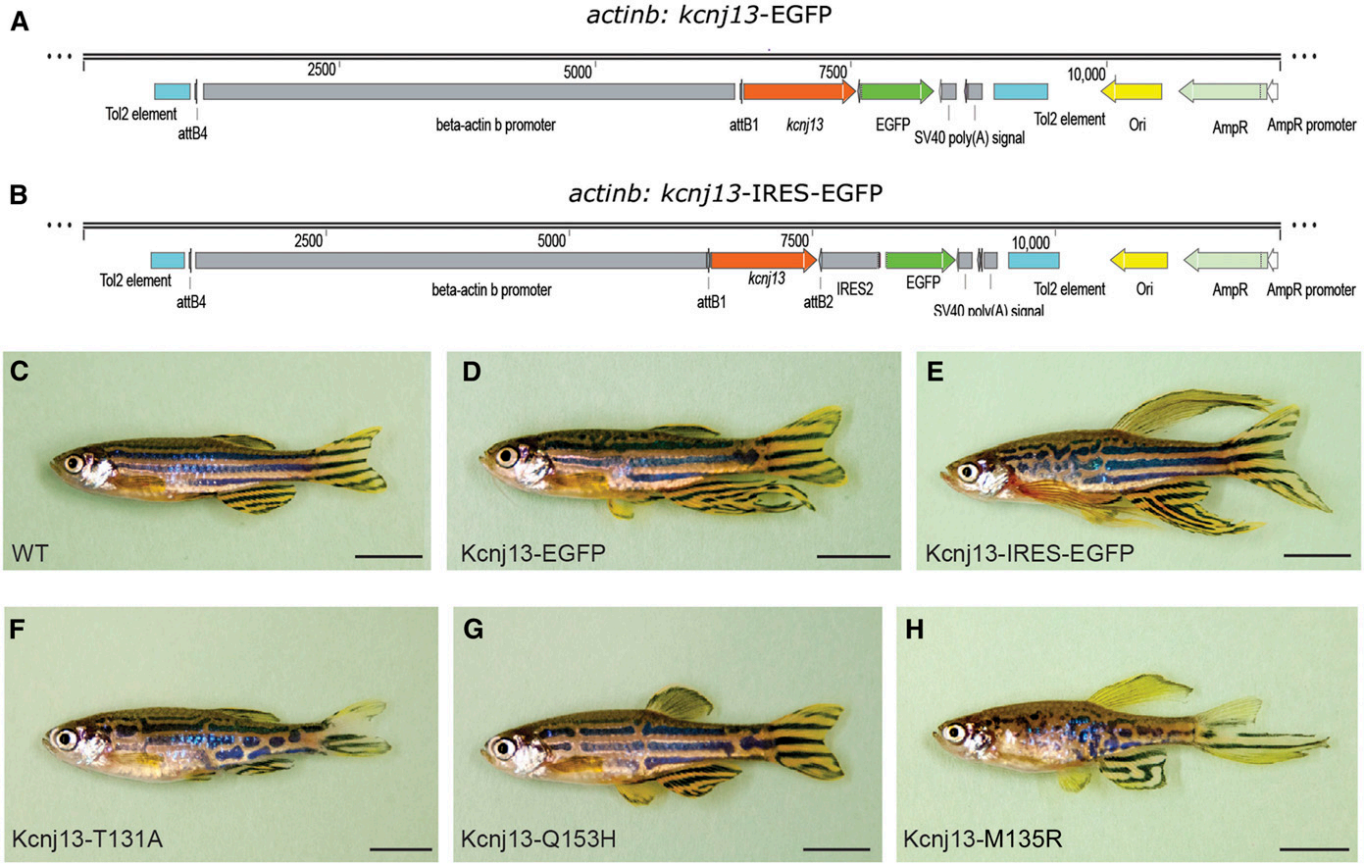
Long-finned phenotype is dependent on potassium conductance. (A–E) Overexpression of *kcnj13* mimics Dhi2059 phenotypes. (A and B) Schematic illustration of constructs used for Tol2 transgenesis with a *kcnj13-EGFP* fusion construct or with independent EGFP. (C) WT noninjected fish. (D) Representative injected fish with *actinb-kcnj13-EGFP* construct. (E) Representative injected fish with *actinb-kcnj13*-IRES-EGFP construct. (F–H) Representative phenotypic changes of the three *kcnj13* mutants. *actinb-kcnj13-T131A-IRES-EGFP* (F), *actinb-kcnj13-Q153H*-IRES-EGFP (G), and *actinb-kcnj13-M135R-IRES-EGFP* (H). Only M135R-, but not T131A- or Q153H-, injected fish developed long-fins, but all have pigmentation pattern disruption. Partial elongation of certain fins and pigmentation changes most likely result from the mosaic nature of Tol2 transgenesis. Bars, 5 mm. AmpR, ampicillin resistance, EGFP, enhanced GFP; IRES, internal ribosome entry site; WT, wild-type.

Subsequently, these three electrophysiologically validated Kcnj13 mutants and wild-type control constructs were injected into single-cell-stage zebrafish embryos. We then raised them to adulthood to check fin sizes. Although the *actinb* promoter is expected to be widely expressed, it would still be able to mediate the ectopic expression of *kcnj13* in the dermomyotome. Indeed, the fins were longer in > 50% of fish injected with either intact Kcnj13 (IRES-EGFP) or Kcnj13 with C-terminal fusions of EGFP (Figure 7, A–E and Table 1). Partially elongated fins in some injected fish (Figure 7, D–H) were most likely due to the mosaicism of the transgene in the injected fish. In contrast, the two less-conductive mutants (T131A and Q153H) failed to induce long-fin formation (Figure 7, F and G, Table 1). However, the more conductive mutant (M135R), similar to wild-type Kcnj13, was able to induce long fins (Figure 7H, Table 1).

**Table 1:** Biological consequences of ectopic expression of potassium channel genes in zebrafish

Gene name	EGFP	Total number of fish	Number of long-finned fish	Long-finned fish (%)	Number of fish with pigmentation changes	Pigmentation changes (%)
*kcnj13*	Fusion	15	7	47	13	87
*KCNJ13*	Fusion	13	8	62	9	69
*kcnj13*	IRES	15	11	73	12	80
*kcnj13-T131A*	Fusion	31	0	0	15	48
*kcnj13-Q153H*	Fusion	10	0	0	7	70
*kcnj13-M135R*	Fusion	20	10	50	15	75
*kcnj10a*	IRES	11	3	27	3	27
*kcnj1b*	IRES	5	1	20	3	60
*kcna1a*	IRES	16	0	0	0	0
*kcnk9*	IRES	31	2	6	0	0

Potassium genes are under the control of a ubiquitous *actinb* promoter in all the constructs. The total number of each injection is somewhat random as numbers of fish eggs vary from each cross; variable fertility and survival rates contribute to the final number. EGFP, enhanced GFP; IRES, internal ribosome entry site.

### The bioelectric signal required for fin ray patterning is conserved in potassium channels

**Figure 8:**
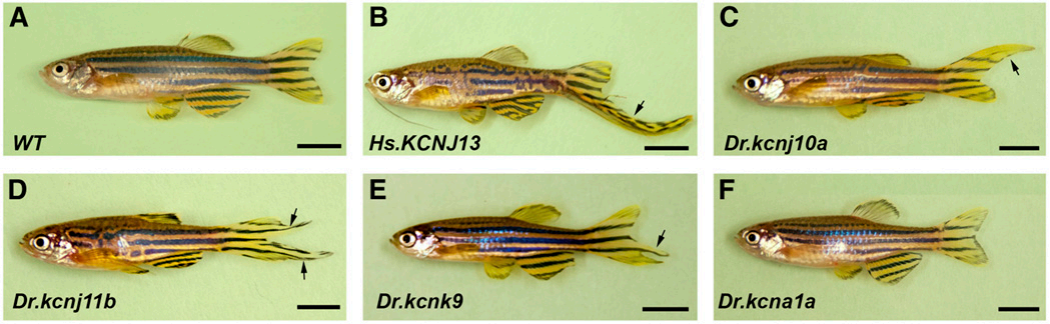
Transient ectopic expression of multiple potassium channel genes also induces long-finned phenotype. (A) WT adult control fish without any injection. (B) Representative injected adult F_0_ fish image of human *KCNJ13*. Note: a barbel is also elongated in this fish. (C) Representative injected adult F_0_ fish image of zebrafish *kcnj10a*. (D) Representative injected adult F_0_ fish image of zebrafish *kcnj1b*. (E) Representative injected adult F_0_ fish image of zebrafish *kcnk9*. (F) Representative injected adult F_0_ fish image of zebrafish *kcna1a*. Arrows indicate the potions of the elongated parts of fins. All the potassium channel genes are driven by the *actinb* promoter. The partial and variable elongated fins are due to the mosaic nature of Tol2 injection. Bars, 5 mm. WT, wild-type.

Our above findings demonstrate that this zebrafish long-finned phenotype depends on Kcnj13 potassium conductance. Potassium conductance is known to influence cell resting membrane potential and other bioelectric properties (Wright 2004). We reasoned that the bioelectricity mechanism for zebrafish fin patterning might not be limited to a specific potassium channel, but instead,a variety of potassium channels may regulate bioelectricity through potassium cellular distribution. To test this hypothesis, we performed *actinb* promoter-driven Tol2-mediated transgenesis experiments with three other transporter Kir channel genes (*kcnj1b*, *kcnj10a*, and human *KCNJ13*), which share a high degree of similarity with *kcnj13*, *kcnk9* (another K2P potassium channel similar to *kcnk5b*), and *kcna1a* (a shaker voltage-gated potassium channel). Indeed, our functional studies demonstrated that long fins were found in most of the transgenic fish except for the fish expressing *kcna1a* (Figure 8 and Table 1). Thus, our results indicate that cellular potassium regulation is the key for fin patterning, and is not necessarily limited to a specific type of potassium channel. Interestingly, we noticed that EGFP expression was not always correlated with elongated fin rays in the adult F_0_ injected fish (Figure S8). The elongated fins may not express GFP, and regular-sized fins can be labeled with GFP. These results suggest that elongated fin rays may not necessarily result from fin-local potassium changes, and that local potassium changes may not be enough to induce elongated fins.

## Discussion

The results presented here demonstrate that transient ectopic expression of the *kcnj13* gene in the dermomyotome underlies the long-finned phenotype in the zebrafish Dhi2059 mutant. The finding that potassium conductance of the Kcnj13 channel is critical for the long-finned phenotype shows that the mutation acts mainly on bioelectric signaling. This conclusion is supported by experiments demonstrating that other potassium channel genes (*kcnj1b*, *kcnj10a*, and *kcnk9*) were able to induce a similar *kcnj13* long-finned phenotype. Thus, our data suggest that dermomyotome bioelectricity is a new fin-patterning mechanism during early embryogenesis. Together with previous data on the fin local bioelectricity from *kcnk5b* and *kcc4a* mutants (Perathoner *et al.* 2014; Lanni *et al.* 2019), we proposed a two-stage bioelectricity model for zebrafish fin patterning.

### The dermomyotome is a new regulatory domain for fin patterning

Zebrafish fin development shares similarities with tetrapod limbs at both the morphological and molecular levels (Iovine 2007; Zuniga 2015). The developmental signaling centers that pattern the endoskeletons of limbs and fins are highly conserved in tetrapods and fish (Iovine 2007; Zeller *et al.* 2009). The zebrafish mutant Dhi2059 has unaltered expression of gene makers of these signaling centers (*shha*, *fgf8a*, and *hoxd13a*) during fin bud development, suggesting that *kcnj13* regulates fin ray development independently of the Shh and Fgf signaling pathways. Previous results on fin development in sharks and lampreys have indicated that the molecular mechanisms responsible for the formation of paired appendages may have evolved in somite-derived tissues (Freitas *et al.* 2006). Recent discoveries, based on long-term cell lineage tracking, have revealed that the zebrafish and medaka dermoskeleton, including fin rays, originate from somite cells (Lee *et al.* 2013a,b; Shimada *et al.* 2013). The somitic origin of zebrafish fin rays is further supported by the discovery that the fourth somite contributes to the formation of the zebrafish larval AEF (Masselink *et al.* 2016). Our findings that ectopic expression of *kcnj13* and other potassium channel genes in the dermomyotome leads to long fins in Dhi2059 mutants and transgenic experiments are consistent with a role for somites in fin ray development. They indicate that the bioelectricity of the dermomyotome is a key patterning mechanism for zebrafish fins.

The transient ectopic expression of *kcnj13* in the dermomyotome might directly influence the fin ray precursor cells within the somite for the first 2 dpf. Dark and bright EGFP F_3_ embryos develop slightly long and quite-long fins once they reach adulthood, respectively. This phenomenon indicates that there is a gene dosage effect on this patterning mechanism. In addition, green fluorescence is absent during the fin regeneration process after fin amputation of adult Tg(*pax3a*-5.4k:*kcnj13-IRES-EGFP*) fish (data not shown), indicating that this dermomyotome mechanism is independent of the previously reported fin-local regulation by *kcnk5b* and *kcc4a* (Perathoner *et al.* 2014; Lanni *et al.* 2019). It is also notable that long fins grew back without *pax3a* promoter-driven *kcnj13* (green fluorescence). This suggests that fin patterning may have already been determined during early development, and the bioelectricity of local fin cells may not be required for fin regeneration. Also, we noticed that EGFP expression was not always correlated with elongated fin rays in the adult fins of F_0_ injected fish. As *pax3a* promoter-driven *kcnj13* ectopic and transient expression occurs in the dermomyotome and the neural crest, we cannot completely rule out the involvement of neural crest cells. Future lineage tracking experiments with zebrafish recombinase lines (Carney and Mosimann 2018), and additional transgenic studies with somite (dermomyotome and sclerotome) and neural crest-marker gene promoters will be helpful for further validation and clarification.

### Bioelectricity is a key mechanism for fin patterning: two-stage bioelectricity regulation model

Our transgenic experiments demonstrated that multiple types of potassium channel produced a similar long-finned phenotype, suggesting that the bioelectric signal required for fin patterning is not restricted to the *kcnj13* gene, but can be mediated through other potassium channels. Previous reports of fin size regulation by *cx43*, *kcnk5b*, and *kcc4a* also support this argument (Sims *et al.* 2009; Perathoner *et al.* 2014; Lanni *et al.* 2019), since all of these proteins are involved in maintaining cellular bioelectric properties. In addition to fin patterning, the *kcnj13* gene is known to be involved in melanocyte cell pattering of *jaguar/obelix* mutants (Iwashita *et al.* 2006; Kondo and Miura 2010) and mouse tracheal tubulogenesis (Yin *et al.* 2018), both of which are mediated by bioelectricity. Thus, it appears that bioelectricity serves as a general patterning mechanism for a variety of tissues and organs.

**Figure 9:**
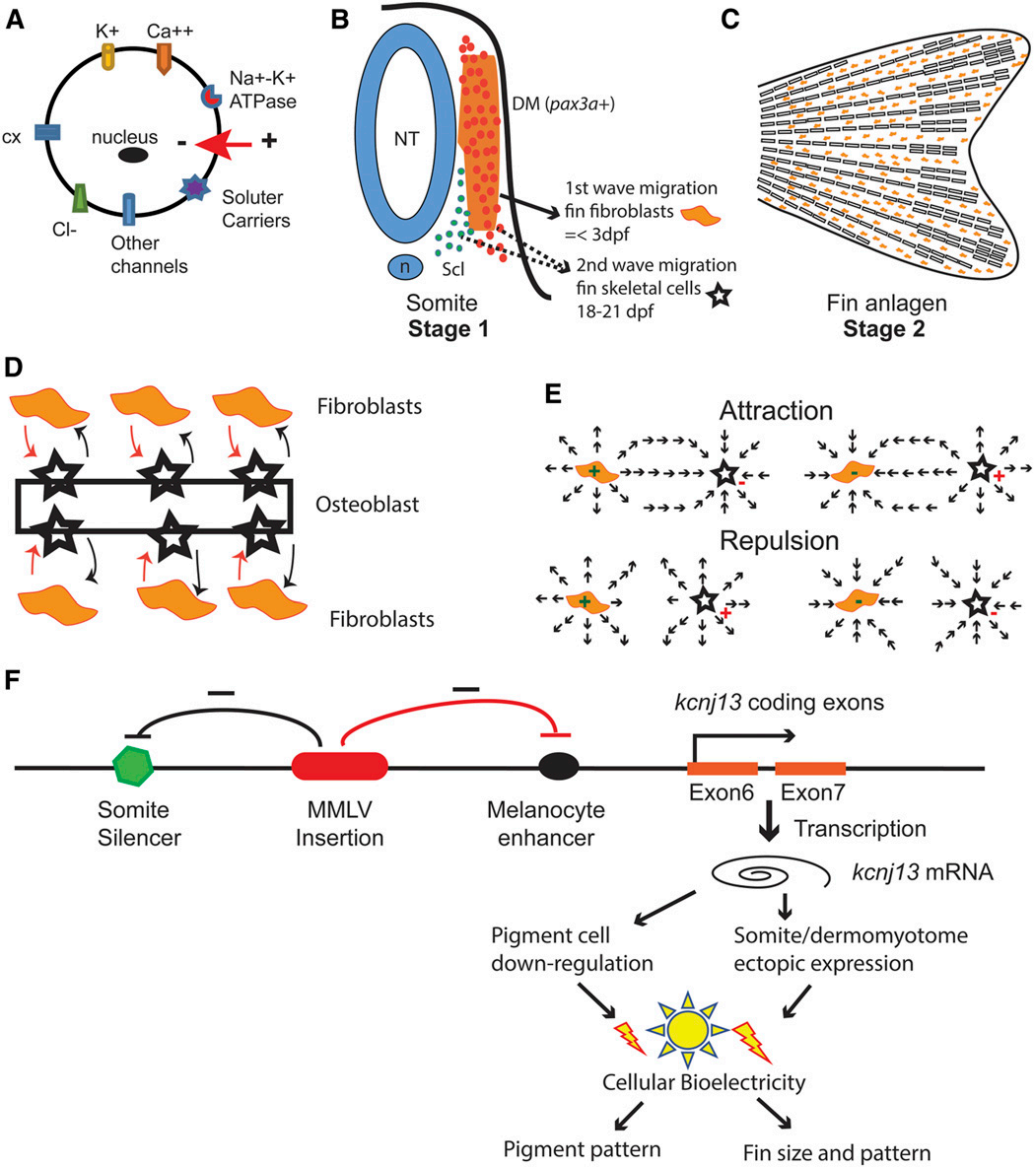
Model of *kcnj13* regulation in the Dhi2059 mutant and fin patterning by bioelectricity. (A) Illustration of cell bioelectric dynamic properties, which may be contributed by potassium channels (K^+^); calcium channels (Ca^++^), chloride channels (Cl^−^), solute carriers, connexins (cx), and Na^+^-K^+^ ATPases. (B–E) Two-stage bioelectric model of zebrafish fins. (B) The first stage of bioelectric regulation happens among the fin progenitor cells within a somite. The dashed lines indicate that the possible source of the second wave progenitors may come from the myotome or sclerotome. The bioelectric alteration of either the first-wave progenitor cells (orange dots) or the second-wave progenitor cells (orange or blue dots) cause fin patterning changes. (C) The second stage of bioelectric regulation happens within the fin anlagen. (D) The interaction of the fibroblasts and osteoblast eventually determine fin size and pattern. (E) Either attraction or repulsion occurs between fibroblasts and osteoblasts, depending on their dynamic bioelectric status. (F) A model of *kcnj13* regulation by retroviral insertion in the Dhi2059 mutant. The Moloney murine leukemia virus is inserted within exon 5 of the *kcnj13* allele in Dhi2059 fish. This viral insertion may negatively affect the melanocyte-specific enhancer and somite/dermomyotome-specific silencer, either through physical distance isolation or viral long terminal repeat regulation. Thus, this insertion leads to ectopic somite expression and slightly reduced expression in the melanocyte. This leads to cell bioelectricity change and subsequent patterning alterations in corresponding organs. DM, dermomyotome; dpf, days postfertilization; mRNA, messenger RNA; n, notochord; NT, Neural tube; Scl, sclerotome.

Based on our current data and evidence from previous reports on the bioelectric regulation of fin growth (Sims *et al.* 2009; Perathoner *et al.* 2014; Lanni *et al.* 2019), we propose a two-stage bioelectric model for zebrafish fin patterning (Figure 9). Multiple ion channels, connexin, and solute carriers may contribute and converge on cell bioelectric properties (Figure 9A). Two waves of somite cell migration are known to contribute to zebrafish fin skeletons. The first wave of cell migration from the dermomyotome leads to fin mesenchymal fibroblast cells (Lee *et al.* 2013b), and the second wave of migrating cells contribute to osteogenesis (Lee *et al.* 2013a). We propose that the bioelectric properties and cell–cell interactions of the progenitors of fin fibroblasts (first wave) and osteoblasts (second wave) are essential for fin patterning. This first-stage regulation is supported by our Dhi2059 mutant and pax3a transgenic experiments. Once the somite progenitor cells move into the fin anlagen and differentiate into fibroblasts and osteoblasts, their interaction through a bioelectric mechanism also influences fin patterning. We refer to this as the second stage of bioelectric regulation (Figure 9, B–D). Previous reports on *kcnk5b* and *kcc4a* are consistent with this, as both were found in fin anlagens (Perathoner *et al.* 2014; Lanni *et al.* 2019). It is possible that the *cx43 short-of-fin (sof)* mutant also falls into this category (Iovine *et al.* 2005). Although the Dhi2059 long-finned phenotype was not rescued by the *kcnj13* jaguar mutant, quantitative measurement revealed that the fin size of the *Kcnj13^Dhi2059/G157E^* mutant was slightly shorter than that of the *kcnj13^Dhi2059/+^* mutant. Thus, these data indicate that Kcnj13 might also function locally in fin anlagens, although we failed to detect any *kcnj13* gene expression in fin anlagens of Dhi2059 fish. In this two-stage bioelectricity model, the dynamic bioelectric properties of the cells may dictate cell behaviors (attract or repel each other), and eventually determine fin pattern and size (Figure 9E). Of course, future studies are needed to test this model. Currently, the specific biochemical components of this bioelectricity pathway remain mostly unexplored. It would be of great interest to identify the bioelectric sensors and mediators downstream of cell membrane potential in the future.

### *Cis*-regulation of ion channels may serve as a driving force for vertebrate morphological diversity, evolution, and human congenital diseases

Teleosts are a successful group of vertebrates with highly diverse fin morphologies and pigmentation patterns (Helfman *et al.* 2009; Nelson *et al.* 2016). How this diversity was achieved during evolution remains largely unknown. However, one of the currently accepted theories is referred to as “a genetic theory of morphological evolution” proposed by Sean Carroll (Carroll 2008). This theory has two major components: (1) morphological form change is mainly caused by gene spatial and temporal expression, and (2) changes of *cis*-regulatory sequences are primarily responsible for such gene expression changes. This notion is supported by the recent manipulation of *hoxd13a* gene expression levels during zebrafish fin development, which causes fin fold reduction and distal elongation of the endoskeleton, mimicking the events thought to have happened during the transition from fins to limbs (Freitas *et al.* 2012).

Although mutants of *kcnj13*, *kcca4*, and *kcnk5b* can induce long fins in zebrafish, the natures of the mutant channel genes are different. Both *kcc4a* and *kcnk5b* are mutants in the protein-coding region, and both function locally in fin anlagens (Perathoner *et al.* 2014; Lanni *et al.* 2019). The Dhi2059 viral insertional mutation is located within the protein noncoding fifth exon and causes the transient and ectopic expression of *kcnj13* during zebrafish early development. This result suggests that multiple *cis*-regulatory elements (CREs) in the gene locus may regulate the activity of the gene temporally and spatially. Indeed, there are five noncoding exons (which encode a long 5′ UTR and 11 uORFs) in the zebrafish locus that are likely to harbor such regulatory elements. The dynamic regulation of *kcnj13* may happen on a translational level through uORFs, which has been reported in many human diseases (Barbosa *et al.* 2013). However, this is unlikely cause tissue-specific changes either through physical distance disruption or retroviral LTR regulation. A more likely case is that there is a tissue-specific silencer that may exist within or around this exon to inhibit *kcnj13* somite expression (somite silencer). The retroviral insertion or LTR may interfere severely with this somite silencer, but only slightly with melanocyte enhancers in the Dhi2059 mutant (Figure 9F). Thus, the Dhi2059 fish have specifically and dominantly affected fin morphology. This is in agreement with the modularity of CREs (Carroll 2008; Douglas and Hill 2014). CREs in other upstream regions and introns may direct *kcnj13* expression in melanocyte cells (melanocyte enhancers). In our heteroallelic analyses, the pigmentation pattern of the double mutant *kcnj13^Dhi2059/G157E^* is similar to that of the *jaguar* homozygotes (*kcnj13^G157E/G157E^*), suggesting that retroviral insertion might inhibit the melanocyte enhancer. Thus, *kcnj13* gene expression is slightly downregulated by this *cis*-regulation, but *in situ* hybridization fails to distinguish *kcnj13^Dhi2059/+^* from wild-type. This subtle change is not enough to alter pigment patterns when the wild-type allele is present, as in the case of *kcnj13^Dhi2059/+^*. However, this change becomes evident in the presence of the *jaguar* mutant allele, due to a dominant-negative effect in the case of *kcnj13^Dhi2059/G157E^*. Considering the recent report on the pigmentation changes in *kcc4a* (*schleier)*, *slc24a5* (*golden*), and connexin mutants (Cx41.8, *leopard*) (Cx39.4, *luchs*) (Lamason *et al.* 2005; Watanabe *et al.* 2006; Irion *et al.* 2014; Lanni *et al.* 2019), pigmentation patterning could also be mediated by bioelectricity through multiple ion channels, connexins, and solute carriers.

Cell membrane potential and other cell bioelectric properties depend on these ion channels, connexins, and solute carriers, which are expressed temporally and spatially during development. Thus, it is reasonable to expect that mutations in the *cis*-regulatory regions of these ion channels may play essential roles in animal morphological evolution and human congenital diseases (Niemeyer *et al.* 2001; Hubner and Jentsch 2002). Along this line, we have also investigated the *KCNJ13* loci of humans and mice, which do not have teleost fish characters, including fin rays and pigmentation stripes. Interestingly, the noncoding exons and long 5′ UTRs are missing, suggesting that these gene regulatory CREs are different between zebrafish and the two mammalian species. Further studies on the developmental mechanisms of bioelectricity and ion channels will shed light on how these biological processes are determined.
